# The Reliability and Validity of a Four-Minute Running Time-Trial in Assessing ${\dot{\text{V}}\text{O}}_{2}$max and Performance

**DOI:** 10.3389/fphys.2017.00270

**Published:** 2017-05-03

**Authors:** Kerry McGawley

**Affiliations:** Swedish Winter Sports Research Centre, Department of Health Sciences, Mid Sweden UniversityÖstersund, Sweden

**Keywords:** graded-exercise test, maximal oxygen uptake, reproducibility, testing, verification phase

## Abstract

**Introduction:** Traditional graded-exercise tests to volitional exhaustion (GXTs) are limited by the need to establish starting workloads, stage durations, and step increments. Short-duration time-trials (TTs) may be easier to implement and more ecologically valid in terms of real-world athletic events. The purpose of the current study was to assess the reliability and validity of maximal oxygen uptake (${\dot{\text{V}}\text{O}}_{2}$max) and performance measured during a traditional GXT (STEP) and a four-minute running time-trial (RunTT).

**Methods:** Ten recreational runners (age: 32 ± 7 years; body mass: 69 ± 10 kg) completed five STEP tests with a verification phase (VER) and five self-paced RunTTs on a treadmill. The order of the STEP/VER and RunTT trials was alternated and counter-balanced. Performance was measured as time to exhaustion (TTE) for STEP and VER and distance covered for RunTT.

**Results:** The coefficient of variation (CV) for ${\dot{\text{V}}\text{O}}_{2}$max was similar between STEP, VER, and RunTT (1.9 ± 1.0, 2.2 ± 1.1, and 1.8 ± 0.8%, respectively), but varied for performance between the three types of test (4.5 ± 1.9, 9.7 ± 3.5, and 1.8 ± 0.7% for STEP, VER, and RunTT, respectively). Bland-Altman limits of agreement (bias ± 95%) showed ${\dot{\text{V}}\text{O}}_{2}$max to be 1.6 ± 3.6 mL·kg^−1^·min^−1^ higher for STEP vs. RunTT. Peak HR was also significantly higher during STEP compared with RunTT (*P* = 0.019).

**Conclusion:** A four-minute running time-trial appears to provide more reliable performance data in comparison to an incremental test to exhaustion, but may underestimate ${\dot{\text{V}}\text{O}}_{2}$max.

## Introduction

Maximal oxygen uptake (${\dot{\text{V}}\text{O}}_{2}$max) testing is widely used to assess aerobic fitness, with procedures typically involving a graded-exercise test (GXT) to volitional exhaustion. While guidelines have been published for common exercise modes such as cycling, running, and rowing (Gore, 2000; Winter et al., 2008; Cooke, 2009), standardized GXT protocols do not exist for individual sports. Instead, starting workloads, stage durations, and step increments all need to be established prior to the test in an effort to produce a final test duration that lies within the recommended range of 5–10 min (Jones, 2008). Open-ended GXT protocols (i.e., without a fixed end-point) lead to variations in test duration that significantly affect the ${\dot{\text{V}}\text{O}}_{2}$max measurement (Yoon et al., 2007) and produce unreliable measures of performance (Currell and Jeukendrup, 2008). As such, alternative protocols may be preferable.

Beltrami et al. (2012) demonstrated higher ${\dot{\text{V}}\text{O}}_{2}$max values with a decremental test (i.e., progressive reductions in treadmill speed, rather than increases) compared with a traditional GXT. However, a decremental test suffers from similar practical drawbacks as an incremental test in terms of needing to select starting workloads, stage durations, and step decrements, as well as being open-ended. Another alternative to the traditional GXT has shown significantly higher ${\dot{\text{V}}\text{O}}_{2}$max values in both cycling and running when “clamping” five, 2-min incremental stages according to rating of perceived exertion (RPE) scores (Mauger and Sculthorpe, 2012; Mauger et al., 2013; Hogg et al., 2015). While this type of protocol does overcome the issues associated with open-ended tests, since there is a fixed end-point, the progressive increase in RPE clamps (starting at 11 then increasing to 13, 15, 17, and finally 20) is still incremental and therefore not ecologically valid in terms of real-world athletic events.

All-out cycling tests lasting between 90 and 180 s have been shown to elicit similar ${\dot{\text{V}}\text{O}}_{2}$max values to those attained during typical GXT protocols (Williams et al., 2005; Burnley et al., 2006; Rossiter et al., 2006; Sperlich et al., 2011; Chidnok et al., 2013). However, all-out tests are characterized by an immediate acceleration phase to peak power output followed closely by a reduction in power output as fatigue ensues (Abbiss and Laursen, 2008). Since individual athletic events lasting >90 s are typically characterized by a pacing component, with individuals self-selecting their distribution of physical effort (Abbiss and Laursen, 2008), all-out tests also lack a degree of ecological validity.

Short time-trials (TTs) may be a preferable alternative to all of the aforementioned ${\dot{\text{V}}\text{O}}_{2}$max protocols since they have a fixed end-point and are self-paced. As well as a higher degree of ecological validity, TTs elicit far lower coefficient of variation (CV) scores for performance compared to time-to-exhaustion (TTE) tests (Jeukendrup et al., 1996; Currell and Jeukendrup, 2008). Previous findings have shown that the ${\dot{\text{V}}\text{O}}_{2}$max attained during a 1-mile (~5 min) running TT performed on a 200-m indoor track (Crouter et al., 2001), a 4-km (~5 min) cycle-ergometer TT (Ansley et al., 2004), and a 600-m (~3 min) skate roller-ski TT performed on a treadmill (Losnegard et al., 2012) was similar to that attained during a standard GXT. In some cases, higher ${\dot{\text{V}}\text{O}}_{2}$max values have been produced during TTs compared with GXT protocols (Foster et al., 1993; McGawley and Holmberg, 2014). Despite these findings, and the similarity between TTs and real-world competition, TT protocols have not become standard procedure for assessing ${\dot{\text{V}}\text{O}}_{2}$max in studies with athlete populations. In addition, the validity and reliability of using laboratory-based treadmill running TTs for assessing ${\dot{\text{V}}\text{O}}_{2}$max and performance have not been investigated.

The aims of the current study were (i) to compare the ${\dot{\text{V}}\text{O}}_{2}$max attained during a standard open-ended GXT to volitional exhaustion on a treadmill (STEP) with that attained during a 4-min, self-paced, laboratory-based running TT (RunTT), and (ii) to assess the reliability of the STEP and the RunTT protocols. It was hypothesized that the ${\dot{\text{V}}\text{O}}_{2}$max attained during the RunTT would be similar to that attained during the STEP and that the RunTT would generate more reliable performance data than the STEP.

## Materials and methods

### Participants

Five males and five females (mean ± SD: age 32 ± 7 years, body mass 69 ± 10 kg) were recruited from local running, triathlon, and multi-sport clubs. During the test period participants were completing 4 ± 1 run training sessions per week as well as a mix of swimming, cycling, paddling, cross-country skiing, and gym training. Immediately prior to the start of the study participants were carrying out high-intensity interval run training weekly or bi-weekly and they competed in a mixture of running and/or multi-sport competitions locally and internationally. Best reported 5- and 10-km run times for the group were mean ± SD: 19.5 ± 1.8 and 40.1 ± 3.3 min, respectively. All participants were fully informed about the study before providing written consent to participate and the study was pre-approved by the Regional Ethical Review Board, Umeå University, Umeå, Sweden.

### Study overview

Each participant visited the laboratory on 11 occasions. On the first visit individuals completed familiarization sessions for the STEP and RunTT. The following 10 visits involved participants completing either a STEP, which included a verification phase (VER) or a RunTT. The type of test completed was alternated on each visit and counterbalanced, such that five participants commenced with the STEP + VER and five commenced with the RunTT.

### Equipment

All run tests were completed on a motor-driven treadmill (Rodby RL 3500, Rodby, Vänge, Sweden) with speed externally controlled by the test leader for the STEP and VER trials, and controlled by the participant during the RunTTs. The treadmill was fitted with a laser system allowing participants to automatically increase or decrease the speed during the RunTTs by moving to the front or the rear of the belt, respectively, maintaining a constant speed otherwise (Swarén et al., 2013). Heart rate (HR) was monitored continuously throughout each trial using a Polar system (RS800CX, Polar Electro Oy, Kempele, Finland) and maximal HR (HR_max_) was calculated as a peak 5-s value. Blood lactate concentration was measured from fingertip blood samples (Biosen 5140, EKF diagnostic GmbH, Magdeburg, Germany). Respiratory variables were measured using a mixed expired air procedure with an ergospirometry system (AMIS 2001 model C, Innovision A/S, Odense, Denmark) equipped with a flow meter. The gas analyzers were calibrated with a high-precision mixture of 16.0% O_2_ and 4.0% CO_2_ (Air Liquide, Kungsängen, Sweden) and the flow meter was calibrated at three rates with a 3-L air syringe (Hans Rudolph, Kansas City, USA). The ${\dot{\text{V}}\text{O}}_{2}$max, ${\dot{\text{V}}\text{CO}}_{2}$, and $\dot{\text{V}}\text{E}$were monitored continuously and ${\dot{\text{V}}\text{O}}_{2}$max-values were calculated and reported as 30-s averages. The associated RER-values were calculated as averages for the same time points as those used to calculate ${\dot{\text{V}}\text{O}}_{2}$max.

### Standardized procedures

Participants reported to the laboratory prior to each trial in a fed state and had abstained from alcohol for at least 24 h prior to testing and from caffeine on the day of the trial. Participants arrived rested and had not completed any intense training on the day before testing. Prior to the first experimental trial (visit 2) participants recorded their diet on the day before and the day of testing and were instructed to consume the same diet prior to all subsequent trials. The 10 experimental trials were completed at the same time of day ± 1 h for each individual, in order to control for circadian variance (Reilly and Brooks, 1982). Following the measurement of body mass in minimal clothing participants performed an individualized 10-min warm-up that was standardized prior to each trial and consisted of 5 min of low-intensity running, 3 × 30-s high-intensity intervals separated by 30 s of low-intensity running and finally 2 min of low-intensity running (Watkins et al., 2017). Three minutes after completing the warm-up participants commenced the STEP + VER or RunTT and HR and expired air were measured continuously. Immediately after completing the test participants reported their RPE (Borg, 1990). Fingertip blood samples were taken 1, 2, 3, and 4 min after each test for the measurement of [LAC] and the highest value was recorded as peak [LAC]. Each of the 10 experimental trials were separated by a minimum of 3 days.

### Incremental step test to exhaustion (STEP)

Participants completed a familiarization of the STEP on their first laboratory visit and the five experimental STEP trials followed the same procedures. Following the 10-min warm-up described previously and a 3-min break participants commenced the STEP at a gradient of 1% and either 12, 13, 14, or 15 km·h^−1^, depending on running ability. The gradient of the treadmill was increased by 1% every minute while speed remained constant and participants, receiving standardized encouragement, continued until volitional exhaustion. No feedback was provided during the test regarding performance (e.g., time or gradient) or physiological (e.g., HR or ${\dot{\text{V}}\text{O}}_{2}$max) responses. Time to exhaustion (TTE) was recorded automatically by the treadmill software. Upon completion of the test participants reported their RPE score and fingertip blood samples were collected.

### Verification phase (VER)

Nine minutes after reaching exhaustion in the STEP participants commenced the VER, which consisted of an additional run test to exhaustion (Midgley et al., 2006; Rossiter et al., 2006). The VER was completed at the same gradient as that reached during the STEP (on that particular day) and at 105% of the STEP speed. Expired air and HR were collected throughout the VER trial and TTE was recorded as the performance variable. Upon completion of the test participants again reported their RPE score and fingertip blood samples were collected.

### Four-minute running time-trial (RunTT)

Approximately 12 min after the end of the STEP familiarization participants completed a familiarization of the RunTT, which followed the same procedures as the subsequent experimental RunTT trials. Following the 10-min warm-up described previously and a 3-min break (or the STEP familiarization and a 12-min break in the case of the familiarization trial) participants commenced the RunTT at a gradient of 1% and a default starting speed that matched each individual's STEP speed. Participants were able to modify the speed of the treadmill immediately by moving to the front of the belt to speed up (with an acceleration rate of 0.5 km·h^−1^·s^−1^) or the rear to slow down (with a deceleration rate of 0.4 km·h^−1^·s^−1^). The runners were able to see their position on the treadmill in relation to the front, middle and rear zones on a screen in front of them and were instructed to control the speed in order to cover as much distance as possible in 4 min, thereby producing a maximal effort. Participants could see elapsed time on the screen but received no performance (e.g., distance covered) or physiological (e.g., HR or ${\dot{\text{V}}\text{O}}_{2}$max) feedback. Standardized encouragement was provided throughout each RunTT, as well as verbal information when only 5 s remained. Distance covered was recorded automatically by the treadmill software.

### Criteria for the attainment of ${\dot{\text{V}}\text{O}}_{2}$max

The following criteria outlined by Cooke (2009) were used to assess the attainment of ${\dot{\text{V}}\text{O}}_{2}$max during the STEP, VER and RunTT tests: (i) a ${\dot{\text{V}}\text{O}}_{2}$ increase of <2.0 mL·kg^−1^·min^−1^ or 3% (i.e., a plateau), (ii) an RER value ≥1.15, (iii) a HR value within 10 beats·min^−1^ of the age-predicted maximum (220-age), (iv) a peak [LAC] of ≥8.0 mmol·L^−1^, (v) an RPE of 19 or 20. The ${\dot{\text{V}}\text{O}}_{2}$max plateau was evaluated over the 1-min period preceding the attainment of the highest 30-s ${\dot{\text{V}}\text{O}}_{2}$max value.

### Data analyses

Data are expressed as mean ± SD unless stated otherwise and the level of significance was set at *P* ≤ 0.05. ${\dot{\text{V}}\text{O}}_{2}$max and peak HR, lactate, RER, and RPE responses were compared for each test type and trial using two-way ANOVAs with repeated measures. Sphericity was checked using Mauchly's test and the Greenhouse–Geisser correction was used when the assumption of sphericity was violated. *Post-hoc* tests with Bonferroni adjustments for multiple comparisons were used to identify pairwise differences. All ANOVA and associated *post-hoc* tests were carried out using the Statistical Package for the Social Sciences (SPSS Inc., Chicago, USA). The intra-individual (trial-to-trial) CV was calculated as SD/mean and intraclass correlation coefficients (ICCs) with 95% confidence intervals (CIs) were calculated across the five repeated trials for both ${\dot{\text{V}}\text{O}}_{2}$max and performance. The bias ± 95% limits of agreement between STEP, VER, and RunTT for ${\dot{\text{V}}\text{O}}_{2}$max were evaluated using the Bland-Altman method with multiple observations per individual (Bland and Altman, 2007). The ICCs and Bland-Altman calculations were carried out using MedCalc statistical software (MedCalc Software, Ostend, Belgium).

## Results

The STEP and VER trials lasted 481 ± 56 s (range: 372–606 s) and 108 ± 16 s (range: 79–148 s), respectively, and distance covered during the RunTT was 1114 ± 90 m (range: 967–1306 m). ${\dot{\text{V}}\text{O}}_{2}$max was attained after 469 ± 59, 107 ± 17, and 227 ± 17 s during the STEP, VER, and RunTT trials, respectively. The frequencies of participants fulfilling the criteria for attaining ${\dot{\text{V}}\text{O}}_{2}$max during trials 1–5 for STEP, VER, and RunTT are displayed in Table 1.

**Table 1:** **The number of participants (out of 10) who fulfilled the criteria for attaining ${\dot{\text{V}}\text{O}}_{2}$max during the five STEP, VER, and RunTT trials (T1–T5)**.

		**T1**	**T2**	**T3**	**T4**	**T5**	**Median**
${\dot{\text{V}}\text{O}}_{2}$ (<2.0 mL·kg^−1^·min^−1^ or 3% increase)	STEP	6	8	4	5	5	5
	VER	0	0	0	0	0	0
	RunTT	7	5	5	6	6	6
RER (≥ 1.15)	STEP	1	2	3	1	1	1
	VER	0	0	0	0	0	0
	RunTT	6	5	4	4	2	4
HR (≤10 beats·min^−1^ of age-predicted max)	STEP	8	6	5	6	7	6
	VER	3	2	3	2	2	2
	RunTT	4	4	4	5	5	4
Peak [LAC] (≥ 8.0 mmol·L^−1^)	STEP	8	8	8	8	6	8
	VER	8	8	8	9	7	8
	RunTT	9	8	9	8	7	8
RPE (19 or 20)	STEP	7	7	7	5	7	7
	VER	7	5	4	7	7	7
	RunTT	6	4	7	5	5	5
≥3 criteria attained	STEP	5	6	5	5	6	5
	VER	3	2	1	2	2	2
	RunTT	8	6	6	6	5	6

### Reliability of ${\dot{\text{V}}\text{O}}_{2}$max and performance

The ${\dot{\text{V}}\text{O}}_{2}$max and performance data for the five STEP, VER, and RunTT trials are shown in Tables 2, 3, respectively, together with the trial-to-trial CV- and ICC-values. The CV and ICC for ${\dot{\text{V}}\text{O}}_{2}$max showed similar levels of repeatability between STEP, VER, and RunTT, while reliability for performance was substantially improved for RunTT vs. STEP and VER, as well as for STEP vs. VER.

**Table 2:** **Mean ± SD of ${\dot{\text{V}}\text{O}}_{2}$max (mL·kg^−1^·min^−1^) for each trial for the incremental test to volitional exhaustion (STEP), the verification phase (VER), and the four-minute running time-trial (RunTT) with associated coefficient of variation (CV) and intraclass correlation coefficient (ICC) data**.

***n = 9***	**T1**	**T2**	**T3**	**T4**	**T5**	**CV (%)**	**ICC (95% CI)**
STEP	58.9 ± 6.0	59.5 ± 7.0	58.4 ± 6.6	59.2 ± 7.2	59.8 ± 7.1	1.9 ± 1.0	0.97 (0.92–0.99)
VER	58.1 ± 6.4	58.8 ± 7.3	57.3 ± 6.7	58.4 ± 6.3	57.8 ± 6.7	2.2 ± 1.1	0.97 (0.92–0.99)
RunTT	57.5 ± 5.6	57.9 ± 6.4	57.8 ± 5.8	57.5 ± 6.1	57.5 ± 6.6	1.8 ± 0.8	0.97 (0.93–0.99)

**Table 3:** **Mean ± SD of performance for each trial for the incremental test to volitional exhaustion (STEP), the verification phase (VER), and the four-minute running time-trial (RunTT) with associated coefficient of variation (CV) and intraclass correlation coefficient (ICC) data**.

	**T1**	**T2**	**T3**	**T4**	**T5**	**CV (%)**	**ICC (95% CI)**
STEP (s)	475 ± 44	488 ± 57	472 ± 61	486 ± 62	483 ± 66	4.5 ± 1.9	0.83 (0.65–0.95)
VER (s)	116 ± 19	110 ± 18	102 ± 16	109 ± 15	104 ± 13	9.7 ± 3.5	0.59 (0.32–0.85)
RunTT (m)	1123 ± 98	1108 ± 101	1117 ± 94	1115 ± 89	1106 ± 85	1.8 ± 0.7	0.95 (0.88–0.98)

### Validity of ${\dot{\text{V}}\text{O}}_{2}$max

**Figure 1:**
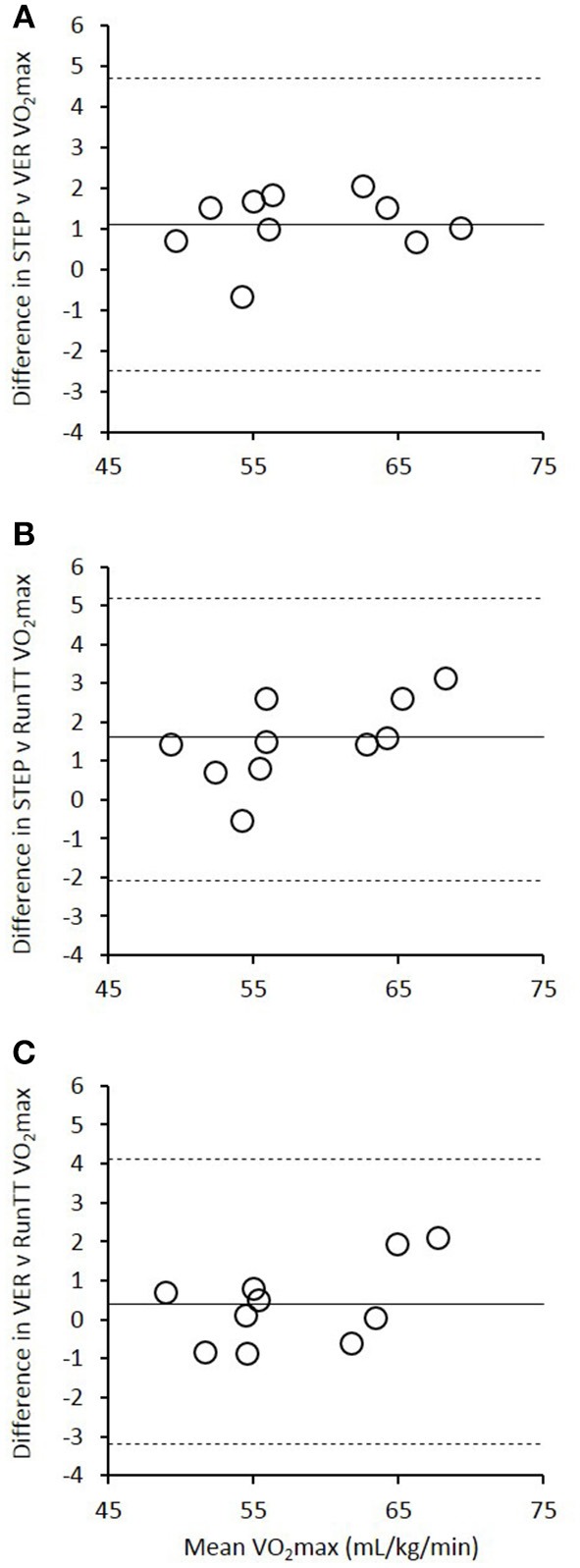
**Bland-Altman limits of agreement (bias ± 95%) for ${\dot{\text{V}}\text{O}}_{2}$max measured during (A)** the incremental test to volitional exhaustion (STEP) and the verification phase (VER), **(B)** the STEP and the four-minute running time-trial (RunTT), and **(C)** the VER and the RunTT.

One missing data point during trial 2 resulted in *n* = 9 for the statistical comparisons associated with the ${\dot{\text{V}}\text{O}}_{2}$max data. There was a significant effect of test type on ${\dot{\text{V}}\text{O}}_{2}$max, with higher values recorded during STEP compared with both VER (*P* = 0.013) and RunTT (*P* = 0.008). However, no significant differences were identified between VER and RunTT (*P* = 0.455). Bland-Altman limits of agreement showed a bias ± 95% of 1.1 ± 3.6 mL·kg^−1^·min^−1^ for STEP vs. VER (Figure 1A), 1.6 ± 3.6 mL·kg^−1^·min^−1^ for STEP vs. RunTT (Figure 1B), and 0.4 ± 3.7 mL·kg^−1^·min^−1^ for VER vs. RunTT (Figure 1C).

### Physiological and RPE responses

**Figure 2:**
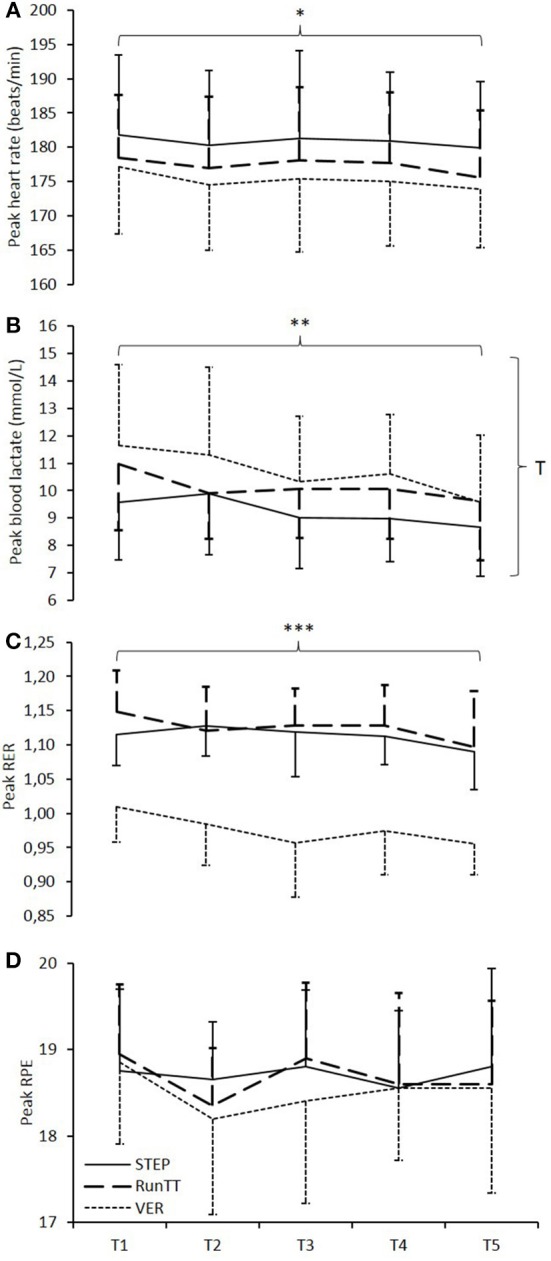
**Peak responses during the five (T1–T5) incremental tests to volitional exhaustion (STEP, solid line), verification trials (VER, dotted line) and four-minute running time-trials (RunTT, dashed line) for (A)** heart rate, **(B)** blood lactate concentration, **(C)** RER, and **(D)** RPE. ^*^STEP significantly higher than VER and RunTT, *P* ≤ 0.05. ^**^STEP significantly lower than VER, *P* = 0.001. ^***^VER significantly lower than STEP and RunTT, *P* ≤ 0.001. ^T^significant trial effect, *P* = 0.039.

There was a significant effect of test type on peak HR (*n* = 6 due to a number of corrupt HR files), with significantly higher values recorded during STEP compared with both VER (*P* = 0.004) and RunTT (*P* = 0.019; Figure 2A). Peak lactate was significantly lower for STEP vs. VER (*P* = 0.001) and there was a significant trial effect, with higher peak lactate values recorded after trial 1 vs. trial 5 (*P* = 0.039; Figure 2B). Peak RER was significantly higher for both STEP and RunTT compared with VER (*P* < 0.001; Figure 2C), while peak RPE did not differ between any of the test protocols or trials (Figure 2D).

## Discussion

The first aim of the current study was to compare the ${\dot{\text{V}}\text{O}}_{2}$max attained during a standard GXT to volitional exhaustion with that attained during a self-paced, laboratory-based running TT. Findings showed that ${\dot{\text{V}}\text{O}}_{2}$max *O*2 max-values attained during STEP were significantly higher than those attained during RunTT. In addition, Bland-Altman limits of agreement showed a general bias of 1.6 ± 3.6 mL·kg^−1^·min^−1^ for STEP vs. RunTT. The second aim of the study was to assess the reliability of the STEP and RunTT protocols. Findings showed similar levels of variation for ${\dot{\text{V}}\text{O}}_{2}$max in both the STEP and the RunTT; however, the RunTT generated more reliable performance data than the STEP.

### Graded-exercise tests vs. time-trials

Traditional GXTs have been criticized in the literature for a number of reasons. Firstly, no standardized protocol exists and selecting different starting workloads, stage durations, and step increments can significantly affect measures of sub-maximal [LAC], peak HR, and maximal power output (Bishop et al., 1998; Kuipers et al., 2003). Secondly, the point of volitional exhaustion in an open-ended test is subjective and variable, which has been shown to lead to variations in ${\dot{\text{V}}\text{O}}_{2}$max (Yoon et al., 2007) and performance (Currell and Jeukendrup, 2008). Thirdly, the ecological validity of a GXT has been questioned, with Noakes (2008) stating that progressive increases in exercise intensity up to a maximum is not how humans “usually” exercise. For these reasons, the current study was designed to evaluate both the reliability and validity of assessing ${\dot{\text{V}}\text{O}}_{2}$max and performance in the laboratory using a more ecologically valid test.

Self-paced maximal efforts lasting ~4 min have been shown to be effective in eliciting ${\dot{\text{V}}\text{O}}_{2}$max across a variety of exercise modes, including indoor track running, cycle ergometry, and treadmill roller-skiing (Crouter et al., 2001; Ansley et al., 2004; Losnegard et al., 2012; McGawley and Holmberg, 2014). Standardizing time rather than distance, while subtly different from a typically distance-based athletic event (Abbiss et al., 2016), allows for direct comparisons between exercise modes. Treadmill running under laboratory conditions was chosen in the current study in order to provide a greater degree of environmental control compared with running in the field (i.e., on a track). Furthermore, the automated treadmill system used in the current study allowed participants to truly self-pace and produce a maximal effort. While a limited number of studies have attempted to validate alternative run-based protocols for the assessment of ${\dot{\text{V}}\text{O}}_{2}$max (Beltrami et al., 2012; Mauger et al., 2013; Hogg et al., 2015; Scheadler and Devor, 2015), only one study has prescribed a truly self-paced running TT (Crouter et al., 2001). In their study, Crouter et al. (2001) concluded that ${\dot{\text{V}}\text{O}}_{2}$max was not different between a traditional incremental test to exhaustion (where the gradient was fixed at 1% and speed was increased every 2 min) and a 1-mile running TT performed on a 200-m indoor track. This is contrary to the findings of the current study where lower ${\dot{\text{V}}\text{O}}_{2}$max scores were demonstrated during self-paced running.

Reasons for the conflicting findings in the current study may be explained by differences in study design and methods of data analysis. Crouter et al. (2001) performed only one incremental test and one, 1-mile TT. This design is similar to the self-paced RPE-clamped running studies, which have concluded from a single comparison that a similar or higher ${\dot{\text{V}}\text{O}}_{2}$max is achieved during a self-paced trial compared with a traditional GXT (Mauger et al., 2013; Hogg et al., 2015). However, the current study involved a series of repeated measures. Interestingly, comparing tests from the first trial only (using a one-way repeated-measures ANOVA with Bonferroni-adjusted *post-hoc* comparisons) would have revealed no significant differences between STEP, VER, and/or RunTT. Therefore, if a series of comparisons, as well as the Bland-Altman calculations, had not been conducted then the conclusion would also have been one of no difference. This suggests that one-off comparisons may not be sufficient to conclude that ${\dot{\text{V}}\text{O}}_{2}$max will be systematically similar (or different) between tests. This is the first study to have compared test protocols over a series of five repeated trials and the significant difference identified between test type, as well as the bias reflected in the Bland-Altman limits of agreement, suggests that ${\dot{\text{V}}\text{O}}_{2}$max may be underestimated in the RunTT compared with the STEP.

Another likely explanation for the differences between previous and current findings relates to the specific nature of the GXT protocol. In the current study a fixed speed was used during the STEP and the treadmill gradient was increased by 1% every minute until volitional exhaustion. This method was selected in an attempt to elicit a true ${\dot{\text{V}}\text{O}}_{2}$max for each individual, with higher values known to occur during uphill versus flat running (Sloniger et al., 1997; Pringle et al., 2002). By contrast, the RunTT was completed at a fixed gradient of 1% and this was chosen to simulate “normal” middle-distance-type racing conditions. This difference in gradient (i.e., an uphill STEP compared with a flat RunTT) may explain the lower ${\dot{\text{V}}\text{O}}_{2}$max in the RunTT. In support of this notion, the similar ${\dot{\text{V}}\text{O}}_{2}$max values observed by Crouter et al. (2001) were measured using a GXT fixed at 1% with increases in speed and a 1-mile TT completed on a flat, indoor running track (i.e., similar gradients between tests). Furthermore, matched gradients during a run-based GXT and an RPE-clamped self-paced test (both 3%) led to similar ${\dot{\text{V}}\text{O}}_{2}$max scores, while a significantly higher ${\dot{\text{V}}\text{O}}_{2}$max was observed during an RPE-clamped self-paced test with a final incline of 11.0 ± 3.2% (Hogg et al., 2015). Additionally, a higher end-exercise gradient during a run-based GXT (~ 10%) compared with a self-paced test at an 8% gradient led to higher ${\dot{\text{V}}\text{O}}_{2}$max scores during the GXT (Scheadler and Devor, 2015). These results highlight the importance of running gradient when comparing ${\dot{\text{V}}\text{O}}_{2}$max values from different types of test. In practice, the choice of gradient may potentially depend upon the specific purpose of the test.

Most previous studies aiming to validate an alternative running test for assessing ${\dot{\text{V}}\text{O}}_{2}$max have performed only *t*-tests or repeated-measures ANOVAs. However, in a recent study Hogg et al. (2015) presented Bland-Altman plots and a bias of ~1 ml·kg^−1^·min^−1^ for the ${\dot{\text{V}}\text{O}}_{2}$max elicited during a GXT compared with a self-paced test, but no significant difference between the means (both tests were completed at a 3% gradient). Despite this, Figure 3 in their study illustrates individual differences that vary by ~16 ml·kg^−1^·min^−1^ (from ~−10 ml·kg^−1^·min^−1^ for one individual to ~6 ml·kg^−1^·min^−1^ for another). These large inter-individual variations in test data indicate an additional need for test–retest reliability analyses, since an unreliable test is of little practical use. The Bland-Altman analyses in the present study used a modified approach (Bland and Altman, 2007), due to multiple observations made per individual (i.e., each participant performed the same test five times). As such, Figure 1 in the current study does not display individual data points, rather shows grouped data for each of the 10 participants. However, when examining the individual data points there was a somewhat improved range of inter-individual differences between the STEP and RunTT ${\dot{\text{V}}\text{O}}_{2}$max values compared to the results of Hogg et al. (2015), with differences between any single comparison of tests ranging from −2.6 ml·kg^−1^·min^−1^ for one individual to +4.9 ml·kg^−1^·min^−1^ for another (i.e., a total range for mean differences of 7.5 ml·kg^−1^·min^−1^). In addition, the CV for ${\dot{\text{V}}\text{O}}_{2}$max in both the STEP and RunTT protocols demonstrated a high level of reproducibility, with mean values for both protocols of <2%. This is lower than the 3.7% CV reported by Mauger et al. (2013) for two GXT trials in a sub-group of five well-trained runners and similar to the 1.4% value reported by Rollo et al. (2008) for three 60-min treadmill-based TTs in a similar group of 10 runners.

### Reliability of time-trial performance

The present study is the first to have investigated the reliability of self-paced treadmill performance using a laser system of this kind and the CV and ICC data reflect high levels of repeatability, particularly in comparison to both the STEP- and VER-tests. This is perhaps not surprising, given that time-trials are known to be more reproducible than TTE-tests (Jeukendrup et al., 1996; Currell and Jeukendrup, 2008). The low CV and high ICC scores for RunTT performance may be explained by the training status of the participants, who were recreationally—to well-trained according to the classification system of De Pauw et al. (2013), and the pre-experimental familiarization procedures, both of which lead to improved reliability (Currell and Jeukendrup, 2008). While a self-paced treadmill of this type is uncommon it is not unique, with other research groups describing similar systems within laboratory settings (Stöggl et al., 2007; Losnegard et al., 2012). With reliable performance data, a range of novel laboratory-based studies are possible using running or cross-country roller-skiing, as opposed to the more common mode of cycling, whereby pacing and intervention strategies may be examined in relation to maximal performance (Andersson et al., 2016; Stocks et al., 2016; Watkins et al., 2017).

### Criteria for the attainment of ${\dot{\text{V}}\text{O}}_{2}$max

While there appears to be no universal agreement regarding the criteria used to identify the attainment of ${\dot{\text{V}}\text{O}}_{2}$max during a GXT (Howley et al., 1995; Midgley et al., 2007), those recommended by the British Association of Sports and Exercise Sciences cited by Cooke (2009) were used in the current study. These include a ${\dot{\text{V}}\text{O}}_{2}$max plateau (measured as an increase of <2.0 mL·kg^−1^·min^−1^ or 3% from the previous minute), a peak RER ≥ 1.15, a peak HR within ≤10 beats·min^−1^ of age-predicted maximum, a peak [LAC] ≥ 8.0 mmol·L^−1^ and an RPE of 19 or 20. The number of participants attaining at least three of these criteria during each of the maximal trials was analyzed and the median value from the five trials was presented in Table 1 for each type of test. The findings support previous suggestions that the traditional criteria for the attainment of ${\dot{\text{V}}\text{O}}_{2}$max are not consistently met during GXT protocols (Poole et al., 2008; Midgley et al., 2009), with a median of only five out of ten participants achieving at least three of the criteria during the STEP in the present study. The count for the RunTT was slightly higher, but still not compelling, with a median of six out of ten participants attaining at least three of the criteria. Consistent with previous findings (Snoza et al., 2016), the most frequently attained criterion was a peak [LAC] ≥ 8.0 mmol·L^−1^ (median: 8 out of 10 participants for both STEP and RunTT), while the least commonly attained criterion was an RER ≥ 1.15.

### Verification phase

In an attempt to validate the attainment of a true ${\dot{\text{V}}\text{O}}_{2}$max, a verification stage has been introduced to GXT protocols involving a shorter, exhaustive test performed at an exercise intensity slightly higher than the final stage of the preceding GXT. Previous studies have shown similar ${\dot{\text{V}}\text{O}}_{2}$max values elicited during a verification phase as in a preceding GXT (Rossiter et al., 2006; Foster et al., 2007). However, results from the current study suggest that the VER may underestimate the ${\dot{\text{V}}\text{O}}_{2}$max measured during the STEP, with a significant difference identified between the two tests and Bland-Altman limits of agreement demonstrating a bias of 1.1 ± 3.6 mL·kg^−1^·min^−1^. Again, higher ${\dot{\text{V}}\text{O}}_{2}$max values attained during the STEP compared with the VER would not have been identified, had five repeated trials not been performed. Results from the present study therefore support the concerns of Midgley and Carroll (2009), who claim that a lack of convincing evidence exists for using standard verification protocols to confirm the attainment of a “true” ${\dot{\text{V}}\text{O}}_{2}$max.

### Conclusion

The current study has shown that ${\dot{\text{V}}\text{O}}_{2}$max may be underestimated during a self-paced running TT performed on a treadmill at a gradient of 1%, when compared with the ${\dot{\text{V}}\text{O}}_{2}$max measured during a GXT performed at increasing gradients until exhaustion. If obtaining a true estimate of ${\dot{\text{V}}\text{O}}_{2}$max is of greatest concern then the RunTT used in the current study would not be recommended. However, if an ecologically valid measure of ${\dot{\text{V}}\text{O}}_{2}$max for running on flat terrain is a priority (i.e., for track or road runners) then the RunTT may be more relevant. If performance is also of interest then a 4-min self-paced running TT provides a more reliable measure (i.e., distance covered) compared with a GXT (i.e., time to exhaustion). In addition, a TT (unlike a GXT) is also able to provide detailed information regarding pacing strategies, as well as the metabolic cost and anaerobic energy contribution during performance (provided a suitable sub-maximal pre-test is completed; McGawley and Holmberg, 2014; Andersson et al., 2016). Therefore, it is important to understand the limitations of each test and select the most appropriate protocol based on the target population and the outcome variables of greatest interest.

## Ethics statement

This study was carried out in accordance with the recommendations of the Regional Ethical Review Board with written informed consent from all subjects. All subjects gave written informed consent in accordance with the Declaration of Helsinki. The protocol was approved by the Regional Ethical Review Board, Sweden.

## Author contributions

KM conceived and designed the study and made substantial contributions to the acquisition, analysis, and interpretation of the data. KM drafted and revised the work, approves the final version to be published, and agrees to be accountable for all aspects of the work.

### Conflict of interest statement

The author declares that the research was conducted in the absence of any commercial or financial relationships that could be construed as a potential conflict of interest.
